# The Relationship between Stressors and Pain-Related Clinical Outcomes in Pediatric Chronic Pain Patients

**DOI:** 10.3390/children8010021

**Published:** 2021-01-04

**Authors:** Anjana Jagpal, Keri Hainsworth, Ratka Galijot, Katherine S. Salamon, Kim Anderson Khan, Susan T. Tran

**Affiliations:** 1 Department of Psychology, DePaul University, Chicago, IL 60614, USA; susan.tran@depaul.edu; 2 Department of Anesthesiology, Medical College of Wisconsin, Wauwatosa, WI 53226, USA; khainsworth@chw.org; 3 College of Osteopathic Medicine, Pacific Northwest University of Health Sciences, Yakima, WA 98901, USA; rgalijot@pnwu.edu; 4 Nemours/AI duPont Hospital for Children, Wilmington, DE 19803, USA; Katherine.Salamon@nemours.org; 5 Children’s Hospital of Wisconsin, Medical College of Wisconsin, Milwaukee, WI 53226, USA; kanderson@chw.org

**Keywords:** chronic pain, stressors, anxiety, functional disability, quality of life

## Abstract

Youth with chronic pain and youth who have experienced stressors are at risk for poor outcomes; however, little is known about the intersection of pain and stressors. This study aims to understand the prevalence of stressors among youth with chronic pain and the relationship between stressors and pain-related outcomes. Seven hundred and seventy youth with chronic pain aged 8–18 (M_age_ = 14.15 years, 70% female) reported pain characteristics, stressors, anxiety, disability, and quality of life. Most participants (82%) endorsed at least one stressor. A greater number of stressors was significantly related to greater anxiety and disability, and lower levels of quality of life. School stressors were significantly associated with functional disability; family, school, and peer stressors were significantly associated with anxiety and quality of life. Stressors are common in youth with chronic pain, and the presence of stressors is related to greater functional impairment. The results of this preliminary study using semi-structured clinical interviews suggest the importance of developing a validated measure that encompasses a wide variety of stressors for youth with pain. Future research on patient-reported stressors, relative intensity, and impact are needed.

## 1. Introduction

It is well established that youth with chronic pain suffer from functional disability and poor health-related quality of life, resulting in emotional distress and anxiety [1,2,3,4]. Although stressors other than pain have the potential to exacerbate these pain-related clinical outcomes, little is known about the prevalence of stressors in youth with chronic pain in addition to their chronic pain, or the effects of these other stressors on functioning. An examination of youth with recurrent abdominal pain found that they experienced greater daily stressors than youth without pain, and that these stressors were related to greater somatic symptoms [5]. Experiencing stressors in general also places individuals at greater risk for anxiety [6] and post-traumatic stress symptoms [6,7]. Stressors disrupt overall functioning leading to poor health-related quality of life and can negatively impact mental health [8,9]. Considering evidence that the experience of multiple stressors can negatively impact physical and mental health in an additive fashion, youth with chronic pain who experience stressors in addition to their pain may be more functionally disabled as a result.

Stress is conceptualized and defined inconsistently in the literature, encompassing both stressors and stress responses. Experts in stress research have proposed an operational definition for stress where “stressor” references the environmental (physical and psychological) events that negatively impact an individual and the term “stress” as a more comprehensive term referring to the environmental events and the consequences of events that develop as a result of exposure to a stressor [10]. This study focused on the reports of a wide range of specific stressors, both physical and psychological, among youth with chronic pain.

Experiencing stressors disrupts the body’s homeostasis and threatens physical and psychological well-being [11]. Repeated exposure to stressors leads to hypothalamic–pituitary–adrenal axis dysregulation. Dysregulation of the stress response systems leads to negative physiological consequences including pain [12]. These negative physiological changes may exacerbate pain and make it more difficult to treat [13]. In addition to the immediate negative physiological consequences, some stressors have a long-term physiological impact, with effects lasting into adulthood [14]. For instance, childhood stressors have been linked to health problems in adulthood such as diabetes, cardiovascular problems, and functional limitations [15]. The relationship between early life stress and long-term health outcomes is complex, likely influenced through behavioral, social, cognitive, and emotional mechanisms [16]. While the impact of these early life stressors has been examined in adults, a better understanding of the effect of a wide range of stressors on pain in youth with chronic pain is needed.

A growing body of research suggests that early life stressors increase the risk of developing and exacerbating chronic pain [5,17] with relationships between environmental stressors and pain complaints manifesting as early as adolescence [18]. However, there is little research on the prevalence of different types of stressors among youth with chronic pain and the impact these stressors may have on pain, as well as key psychological and functional outcomes. Recent findings from nationally representative samples showed that exposure to adverse childhood experiences (ACEs) and community violence was related to a higher incidence of chronic pain [18,19]. Similarly, research has demonstrated that youth with chronic pain experience high rates of ACEs and stressful life events [19,20,21]. Furthermore, ACEs and post-traumatic stress disorder (PTSD) symptoms among youth with chronic pain are related to greater physical symptomatology, and PTSD symptoms are related to worse health-related quality of life and greater pain interference [20,21]. While there is conceptual overlap between ACEs, PTSD symptoms, and stressors, it is important to consider that “stressors” can include a number of other events not included in the previously mentioned categories (i.e., social and academic stressors). Furthermore, stressors, major and minor, rarely occur in isolation, and it is important to consider the breadth of stressors experienced by adolescents with pain. Therefore, the current study adds a unique perspective by examining the relationship between a wide range of stressors and important pain-related clinical outcomes. Because youth with chronic pain commonly experience poor outcomes such as anxiety, functional disability, and poor quality of life (e.g., [1,3,22]), it is important to investigate whether stressors exacerbate these negative outcomes.

The current study aimed to explore the frequency and nature of stressors experienced by youth with chronic pain and examine the effects of stressors on pain-related clinical outcomes (pain, anxiety, functional disability, and quality of life). It was hypothesized that youth with chronic pain who endorsed a greater number of stressors would have worse pain-related outcomes than those with fewer stressors. Finally, the relationships between specific categories of stressors and pain-related outcomes were explored to determine whether specific stressors are uniquely related to different domains of functioning. As the nature of specific categories of stressors and their impact on functioning has not been examined previously, no specific hypotheses were tested in these exploratory analyses.

## 2. Materials and Methods

### 2.1. Participants

Participants were 770 youth ages 8–18 years who presented to a chronic pain clinic at a large Midwestern children’s hospital in the United States. Inclusion criteria included (1) chronic or recurrent pain present for 3 months or longer, (2) 8–18 years of age, and (3) ability to read and comprehend English. Participants were excluded if they did not arrive to their appointment early enough to complete the measures, if they did not speak English or had significant developmental delay that would hinder their ability to respond on self-report measures. The current study was approved by the Institutional Review Board of the children’s hospital (IRB#615713).

### 2.2. Procedures

A packet of questionnaires was mailed to the participants to complete at home prior to their visit. If the measures were not filled out beforehand, participants were invited to complete study questionnaires at the time of their visit then they met with a pain physician and psychologist for an intake appointment at a multidisciplinary pain clinic located within a children’s hospital. Each participant was assigned a study identification number, and the research data were entered in a secure database.

### 2.3. Measures

The following demographic information was collected from participants: age, sex, ethnicity, pain locations, and duration of pain.

#### 2.3.1. Pain

The Pain Frequency-Severity-Duration Scale [23] includes questions assessing the patient’s usual pain over the last two weeks using a Likert scale ranging from 0–10 (“No pain” to “Worst pain”) and pain duration. Pain intensity and duration in months was derived from the responses on this measure.

#### 2.3.2. Stressors

A list of 23 stressors was generated by the clinical team and referred to by all psychologists during the clinical interview at the intake appointment. The list included potential stressors based on the team’s collective clinical experience. The list of stressors included family-related factors (e.g., family conflict), school problems (e.g., academic problems), and peer relational stressors (e.g., bullying). During the interview, patients were asked about their functioning across several different domains. The pain psychologist noted each stressor that was endorsed by the patient during the interview. For example, if a patient was asked about school functioning, and they stated that they typically receive high grades and pain was interfering with their ability to do homework, then “high achiever/straight As” was marked. Information from the patient regarding stressors was gathered over the course of the clinical interview and the stressors were measured as a binary variable (endorsed or not endorsed).

#### 2.3.3. Anxiety

The Screen for Child Anxiety-Related Emotional Disorders (SCARED) is a 41-item self-report measure assessing for the presence of clinically significant symptoms of anxiety in youth based on the DSM-IV-TR [24]. The measure is validated for children 8 years and older and is found to be reliable in a pediatric chronic pain sample [25]. Participants report the frequency of anxiety symptoms in the past three months with response options including: 0 = Not True, 1 = Sometimes True, and 2 = Often True. Total scores range from 0–82 with higher scores indicating greater levels of anxiety. Scores of ≥ 25 indicate clinically significant anxiety. The SCARED had good reliability in this study’s sample (α = 0.94).

#### 2.3.4. Functional Disability

The Child Activities Limitations Questionnaire (CALQ) is a valid and reliable 21-item self-report measure of functional disability [26]. Participants report the difficulty of specific activities (i.e., watching T.V., running, walking upstairs, and sleeping) in the past four weeks with response options ranging from 0 “Not at all Difficult” to 5 “Extremely Difficult.” The CALQ had good reliability in this study’s sample (α = 0.89)

#### 2.3.5. Quality of Life

The Pediatric Quality of Life Inventory Version 4.0 (PedsQL) is a valid and reliable 23-item self-report measure assessing quality of life of youth with health conditions [27]. The PedsQL assess the following domains: physical functioning, emotional functioning, social functioning, and school functioning. All domains contribute to a total quality of life score. Participants were given the appropriate measure depending on their age (Child Self-Report for ages 8–12 or Child Self-Report for ages 13–18). A 5-point response scale was utilized (0 = never a problem; 1 = almost never a problem; 2 = sometimes a problem; 3 = often a problem; 4 = almost always a problem). Items are reversed scored and linearly transformed to a scale 0 to 100, where higher scores indicate greater quality of life. The PedsQL had good reliability in the study’s sample (α = 0.90).

### 2.4. Analysis Plan

Data were analyzed using SPSS version 27 [28]. All data were normally distributed. Descriptive statistics were conducted to describe the sample and the stressors endorsed by participants. Descriptive data on demographic characteristics and pain locations were derived and examined in relation to number of stressors.

**Figure 1:**
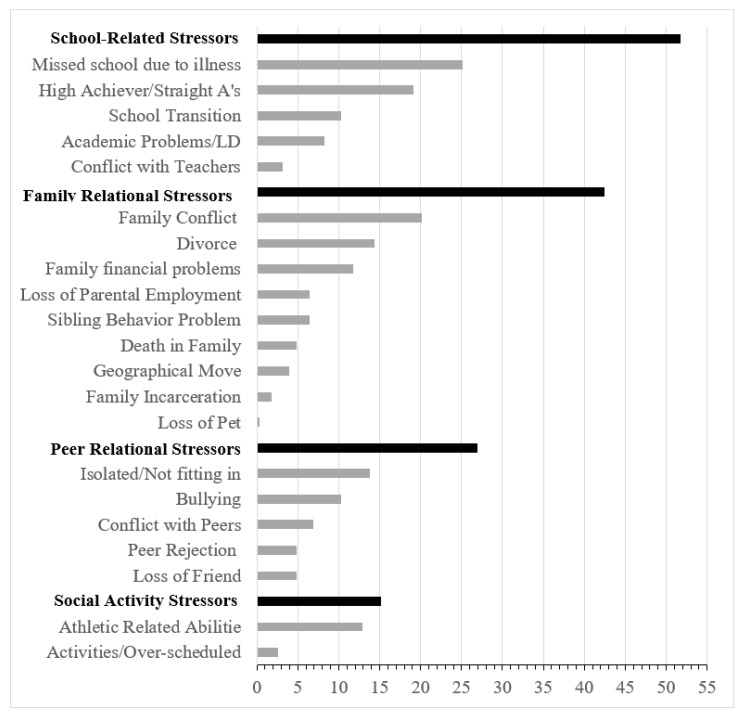
Percentage of participants who reported each stressor. Stressor categories are in bolded rows and include the individual stressors listed below them. Note. Items are not mutually exclusive—participant can report more than one stressor.

Individual stressors were grouped into meaningful categories using the Delphi technique. The Delphi technique is a method in which researchers with a specific area of expertise independently group similar items into meaningful categories [29]. Five research team members, who were different than the clinical team members, independently categorized the 23 individual stressors. The team discussed any discrepancies as a group and came to a consensus about the final categories. Two items were ultimately removed from the dataset (abuse and domestic violence), as these stressors may have different impacts on children compared to other stressors on the list. The final four stressor categories included 21 individual stressors: family relational stressors, school-related stressors, social activity stressors, and peer relational stressors. Stressor items are listed under their respective category in Figure 1.

Hierarchical regressions were performed to examine the impact of stressors on pain-related outcomes beyond that associated with pain alone. The hierarchical regression model included usual pain in the first step and total number of stressors in the second step. Dependent variables included anxiety, functional disability, and quality of life. Total scores for all continuous variables were used. Additionally, ANOVAs were conducted to examine whether or not a dose–response relationship existed between the number of stressors (0, 1, 2, 3, and 4 or more stressors) and pain-related clinical outcomes.

Finally, exploratory hierarchical regressions were performed in order to understand the extent to which the number of stressors in each category of stressors were associated with outcome variables beyond that associated with pain alone. The hierarchical regression model included usual pain in the first step and the four categories of stressors in the second step. Dependent variables included anxiety, functional disability, and quality of life.

## 3. Results

Of the 770 participants in the sample (age 8–18, M_age_ = 14.15, SD_age_ = 2.40), the majority were Caucasian (*n* = 610, 80%) and female (*n* = 542, 70%), which is congruent with other pediatric pain populations [27]. Participants reported the average duration of the primary pain problem to be 28.27 months (SD_duration_ = 29.77, range 3–204 months); the usual pain intensity was M_intensity_ = 6.35 (SD_intensity_ = 2.02, range = 0–10). Common pain locations included: head (41%), trunk (17%), and extremities (17%). Regarding the relationships of stressors with clinical and demographic characteristics, only pain intensity was significantly and positively related to the number of stressors (*r* = 0.11, *p* < 0.01).

Frequency analyses were conducted to examine the relative frequency of all stressors and stressors in each category. The number of stressors reported ranged from 0–9 (M = 1.95) with the majority of the sample (81%) endorsing at least one stressor. Almost 28% of the sample reported one stressor, 24% reported two stressors, 13% reported three, and 17% reported four or more. Overall, the most commonly reported stressor categories were school-related stressors (51.7%), followed by family relational stressors (42.5%), peer relational stressors (26.9%), and social activity stressors (15.2%). Frequencies of individual stressors and categories of stressors are shown in Figure 1.

### 3.1. Relationships Between Stressors and Pain-Related Outcomes

For each of the outcome variables (anxiety, functional disability, and quality of life), a regression analysis examined the contribution of number of stressors to the outcome above and beyond pain. Additionally, ANOVAs were conducted to examine the effects of 0, 1, 2, 3, and 4 or more stressors. Finally, regression analyses examined the relative contributions of the different types of stressors on each outcome variables.

#### 3.1.1. Anxiety

After controlling for pain, a greater number of stressors was significantly related to greater total anxiety scores (*R*^2^ = 0.14, β = 0.33, *p* < 0.001). Additionally, a dose–response effect was examined through an ANOVA; anxiety scores generally increased with additional stressors (F (4657) = 18.74, *p* < 0.001), and individuals with zero stressors had significantly less anxiety than those with two, three, or four or more stressors. Finally, hierarchical regression analysis was utilized to understand the extent to which different stressor categories contributed to the impact on anxiety beyond pain level. Usual pain was significant (*R*^2^ = 0.039, *p* < 0.001). An additional 16% of the variance in anxiety was attributed to stressors entered into the second step, and all of the stressor categories were significantly related to anxiety (see Table 1).

#### 3.1.2. Functional Disability

Participants with a greater number of stressors had significantly greater functional disability scores above and beyond pain intensity (*R*^2^ = 0.19, β = 0.09, *p* = 0.01). Additionally, a dose–response effect was examined through an ANOVA. As functional disability scores generally increased with each additional stressor (F (4683) = 4.12, *p* = 0.003), individuals with zero stressors had significantly less functional disability than those with two, four, or more stressors. Finally, results from the hierarchical regression revealed that pain accounted for 18% of the variance in disability, and stressor categories accounted for an additional 3% of the variance in disability. Of the stressor categories, school stressors were significantly associated with disability (*p* < 0.001) (see Table 1).

#### 3.1.3. Quality of Life

Participants with a greater number of stressors had significantly lower total quality of life scores after controlling for pain (*R*^2^ = 0.18, β = −0.23, *p* < 0.001). Additionally, a dose–response effect was examined through and ANOVA where quality of life scores generally decreased with additional stressors (F (4759) = 12.68, *p* < 0.001), and individuals with zero stressors had significantly higher quality of life than those with two, three, or four or more stressors. Finally, with regard to stressor categories, pain intensity accounted for 13% of the variance in quality of life, and stressors accounted for an additional 8% of the variance in quality of life. All four stressors categories (family, school, social, and peer stressors) significantly predicted quality of life (see Table 1).

## 4. Discussion

The current investigation revealed that stressors are common amongst youth with chronic pain with 82% of this sample reporting at least one stressor. Given the paucity of literature examining the impact of general stressors on youth with chronic pain, the current study adds a meaningful contribution to the literature by highlighting the prevalence of stressors experienced by youth with chronic pain as well as the relationship between stressors and functioning. There are a number of studies examining the negative consequences of specific types of life stressors (e.g., [19,20,30]), and our study supports those previous findings and provides support for the use of a stressors inventory in a pediatric chronic pain setting. Findings expanded on the existing literature on stress and pediatric pain by examining a variety of different types of stressors experienced by youth with chronic pain (e.g., school-related and family-related stressors) and the varied negative outcomes related to stressors. There is room for improvement with regard to the assessment of stressors for future studies to consider; however, our results provide strong preliminary evidence for differential associations between different types of stressors and areas of functioning in a large sample of treatment-seeking youth with chronic pain.

The results indicate that across several domains, youth with chronic pain who endorse experiencing a greater number of stressors are more impaired. Specifically, a dose–response relationship was present across all outcomes where an increasing number of stressors was significantly associated with anxiety, functional disability, and significantly lower quality of life in youth with chronic pain. These findings indicate a potentially low threshold (two or more stressors) for a significant negative impact on pain-related outcomes. The dose–response effect of stressors on anxiety level was quite striking. Given research on the negative impact anxiety has on treatment outcomes in a pediatric chronic pain sample [31], it is likely that the presence of stressors may interfere with progress in treatment for chronic pain if left unaddressed. The impact of chronic pain alone on clinical outcomes (e.g., functional disability, quality of life) is well documented [1,2,3,4]. Therefore, it is noteworthy that stressors may further exacerbate the poor outcomes in youth with chronic pain. One possible explanation for this relationship is that experiencing stressors in addition to chronic pain may over-burden an individual’s coping resources and make coping with chronic pain more difficult.

While there was variability in the types of stressors endorsed by participants in this study, the most commonly reported stressor category was school-related stressors. This finding is consistent with the broader literature, in which school-related disability is one of the most important functional outcomes with respect to pediatric chronic pain [32]. Given the strong relationship between chronic pain and school functioning, it makes sense that school-related stressors were associated with all three outcomes. These findings further emphasize the vulnerability of this age group to school-related stress. Somatic complaints, such as chronic and recurrent pain, are commonly experienced by the time a child reaches adolescence, and school-related stressors often contribute to the maintenance of these symptoms [33]. This relationship is bidirectional in the sense that school-related stress has been linked to an increased risk for chronic pain [33], and pediatric chronic pain is associated with excessive school absenteeism [34,35,36]. Not surprisingly, the most commonly reported stressor in our sample was missed school due to an illness. This is consistent with other findings that youth with chronic pain miss close to 25% of the school year [37]. Furthermore, anxiety is often a driver of school-related disability, which can exacerbate school-related avoidance [38]. There appears to be a negative cycle in which youth with chronic pain have greater levels of school-related anxiety [39], anxiety drives school avoidance, missing school and school-related stressors heighten anxiety [40], and anxiety decreases the ability to manage chronic pain [41,42].

The second most common stressor was family conflict, which was endorsed by 20.1% of the sample. While the literature on family conflict in pediatric chronic pain is mixed [43], our findings are consistent with research suggesting that youth with chronic pain have greater family conflict compared to healthy peers [43]. Specifically, findings have demonstrated higher levels of family conflict and lower levels of family cohesion in a chronic pain sample [44]. While school stressors were associated with all three outcome domains, family relational stressors were associated with anxiety and quality of life, but not functional disability. It is possible that youth with family relational stressors, equipped with appropriate coping strategies, experience reduced quality of life and greater anxiety, while having to seek out other sources of support in friends or teachers, thus functional disability is less impaired.

This study showed that the types of stressors were differentially associated with outcome variables. All stressor categories were significantly related to anxiety, together explaining 15% of the variance above and beyond that explained by pain alone. Findings echo previous research, which established a link between life stressors and anxiety disorders [45]; our findings further suggest that several types of stressors influence anxiety. It is important to pay attention to factors that heighten anxiety, given that anxiety interferes with treatment outcomes for youth with chronic pain [31] and independently increases functional disability [46]. Thus, the source of stressors contributing to poor outcomes in mental and physical health is critical. Family, school, and peer stressors were all related to quality of life. Quality of life is a broader measure, inclusive of mental health functioning, and is likely influenced by a number of factors, thus it makes sense that stressors across domains would contribute to this outcome. Quality of life is also inherently affected by pain, which was significantly associated with quality of life; therefore, it is possible that the presence of stressors exacerbates the impact of pain on quality of life. Finally, pain intensity and school-related stressors are associated with functional disability. School has been called the “work of childhood”, and stressors in this domain are likely directly related to how well a child functions in day-to-day life.

There are several limitations and subsequent future directions that should be noted. A validated stressor inventory was not utilized, the presence of stressors was recorded by clinicians during the clinical intake rather than self-reported, and the list of 23 stressors is not comprehensive. Nonetheless, in lieu of a validated measure, the use of a single set of stressors standardized across the assessment of participants in this study strengthens the results. There is no particular stressor inventory widely used in pediatric chronic pain and most stress studies do not use a validated stressor inventory [47]. It is imperative for future work in this area to develop a comprehensive, psychometrically sound stressors measure assessing a broad array of stressors to better understand the prevalence of stressors amongst youth with chronic pain. It is possible that this population experiences unique stressors relative to their healthy peers, thus future research should explore stressors experienced by adolescents with and without pain and seek patient feedback on measure items through focus groups. Assessment of stressors could be conducted both verbally and through written response in order to account for differences in communication abilities and reporting styles. Additionally, although meaningful groups were developed from the stressors in this study, there are other domains that need to be addressed. For example, community-level stressors (neighborhood environments and community violence) might be related to the pain experience [48]. A validated measure would need to determine whether any endorsed stressors are in the past or ongoing, or if the stressors preceded or followed the onset of pain, at the time of the assessment. The current study examined important clinical outcomes including anxiety, functional disability, and quality of life. An important consideration is that youth with anxiety are likely to view situations or experiences as more stressful compared to their non-anxious counterparts. Future work should better understand if anxiety is predictive of more stressors and subsequent poor clinical outcomes in a pediatric chronic pain sample. Lastly, the cross-sectional research design limits the ability to understand the long-term impact of stressors and pain and the bi-directional relationship between pain and stressors over time. There is well-documented literature on the negative impact of chronic or repeated stress; therefore, future work should explore the long-term implications of chronic stress on youth with chronic pain. For example, variations in the stress response and the relationship to functioning is another area warranting research.

## 5. Clinical Implications

The results of the current study highlight the high prevalence of stressors in this population and suggest the importance of assessing for the presence and the impact of these stressors in youth presenting to pediatric chronic pain clinics. Future research is needed to develop and validate an inventory of stressors for youth with chronic pain to better assess for factors, which may be related to functional outcomes. With improved understanding of the impact of stressors in this population, it may be necessary to address those stressors in treatment. This may mean adding additional targets for treatment (e.g., improving family communication) or incorporating coping skills to address stressors other than pain. Finally, all domains of stressors examined were significantly related to anxiety; thus, the results of this study support previous calls for increased attention to sources of anxiety and appropriate treatment of anxiety in youth with chronic pain [41,42,46,49].

## 6. Conclusions

Stressors are common in youth with chronic pain, and they have a negative effect on functioning across domains. There may be specificity in the types of stressors that impact certain domains of functioning (i.e., significant impact of school and peer stressors on anxiety and quality of life and the unique impact of school stressors on disability). A better understanding of the mechanisms mediating the relationships between specific stressors and outcomes in youth with pain is necessary, because it will help in understanding the role of pain-related stress and identifying possible targets for intervention.

## Figures and Tables

**Table 1:** Prediction of pain-related outcomes with stressor categories.

	Anxiety					Functional Disability					Quality of Life				
	B	SE	β	Δ*R*^2^	F	B	SE	β	Δ*R*^2^	F	B	SE	β	Δ*R*^2^	F
**Step 1:**				0.04	26.19 ***				0.18	144.72 ***				0.13	111.44 ***
Usual Pain	1.45	0.28	0.20 ***			4.74	0.39	0.42 ***			−3.15	0.30	−0.36 ***		
**Step 2:**				0.16	31.49 ***				0.03	33.95 ***				0.08	38.58 ***
Family	2.50	0.53	0.17 ***			−0.12	0.82	−0.01			−1.58	0.59	−0.09 **		
School	1.81	0.71	0.09 *			4.37	1.09	0.14 ***			−3.17	0.80	−0.13 ***		
Social	−4.00	1.37	−0.10 **			−3.14	2.12	−0.05			3.85	1.56	0.08 *		
Peer	5.09	0.66	0.28 ***			1.19	0.99	0.04			−3.79	0.74	−0.17 ***		

Note. * *p* < 0.05, ** *p* < 0.01, *** *p* < 0.001.

## Data Availability

The data presented in this study are available on request from the corresponding author. The data are not publicly available due to confidentiality agreements.
